# How TAVI registries report clinical outcomes—A systematic review of endpoints based on VARC-2 definitions

**DOI:** 10.1371/journal.pone.0180815

**Published:** 2017-09-14

**Authors:** Shixuan Zhang, Peter L. Kolominsky-Rabas

**Affiliations:** 1 Interdisciplinary Centre for Health Technology Assessment (HTA) and Public Health (IZPH), Friedrich-Alexander University of Erlangen-Nürnberg, Erlangen, Bavaria, Germany; 2 National Leading-Edge Cluster Medical Technologies “Medical Valley EMN”, Erlangen, Bavaria, Germany; Universita degli Studi di Roma La Sapienza, ITALY

## Abstract

**Introduction:**

Transcatheter aortic valve implantation (TAVI) has been demonstrated to be an alternative treatment for severe aortic stenosis in patients considered as high surgical risk. Since its first human implantation by Cribier et al., TAVI has been shown to increase survival rate and quality of life for high surgical risks patients. The objective of this study is to provide an overview of TAVI registries and the reporting clinical outcomes based on the VARC-2 definitions. In addition, the comparability and adherence of VARC-2 reporting within the identified TAVI registries was reviewed.

**Materials and methods:**

A systematic review of TAVI registries reporting VARC-2 definitions has been performed in line with PRISMA guidelines in PubMed, ScienceDirect, Scopus databases and EMBASE. Based on VARC-2, patients’ characteristics and procedure characteristics, 30-day clinical outcomes, 1-year mortality and composited endpoints were extracted from each registry’s publications.

**Results:**

This review identified 466 studies that were potentially relevant, and 20 TAVI registries reported VARC-2 definitions involved in our present review. Of all 20 registries, an overall sample size of 12,583 patients was involved. The 30-day all-cause mortality ranged from 0 to 12.7%. From 20 registries, 14 registries reported the cardiovascular mortality at 30 days. 9 registries reported myocardial infarction (MI) rate based on VARC-2 definitions, and 7 registries reported peri-procedural MI rate (<72h). In our review, most of registries presented MI rates ranging from 0.5% to 2%. The majority of registries have reported complications such as bleeding, vascular complications and new pacemaker implantation.

**Conclusion:**

Since the introduction of VARC definitions from 2011, VARC and VARC-2 definitions are still not systematically used by all TAVI studies. These endpoint definitions warrant a concise and systemic analysis of outcome measures. Reporting TAVI-outcome uniformly makes study result comparison feasible. This definitely will increase patient safety, additionally to provide sufficient evidence to support decision makers like regulatory bodies, HTA agencies, payers.

## 1. Introduction

### 1.1 Rational

The aortic stenosis (AS) is the most common valvar heart disease in developed countries [1], which affects 2% of the population aged 65 years or older [2]. Open surgical aortic valve replacement (SAVR) is the standard care in the treatment of symptomatic AS patients [3]. However, about 30% to 50% of patients with severe AS do not undergo surgery for a variety of high surgical risks such as age and comorbidities [4]. Transcatheter aortic valve implantation (TAVI) has been demonstrated to be an alternative treatment for severe AS in patients considered as high risk for SAVR [5,6].

Since its first human implantation by Cribier et al. [7], TAVI has been shown to increase survival rate and quality of life for high surgical risks patients [8,9]. However, the fast growth of TAVI has created difficulties in cross-study result comparison. Since the investigators were not prepared to interpret clinical data in a standardized way, it made clinical data reporting difficult [10]. In October 2011, the first European consensus document on TAVI, called Valve Academic Research Consortium (VARC), with standardized definitions on clinical endpoints, was published [10]. The goals of VARC are combining the expertise to arrive at a consensus for selecting appropriate clinical endpoints and standardizing definitions for single and composite clinical endpoints [10]. Two years later, it has been subsequently revised in the VARC-2 definitions [11]. The VARC-2 definition is an updated version from the VARC definition. It clearly indicated that all-cause mortality, cardiovascular and non-cardiovascular mortality should be reported after 30 days. In addition, for the major complications, the VARC-2 revisited the selection and definitions of TAVI-related clinical endpoints to make them more suitable to the present and future’s needs of clinical trials. The VARC-2 definitions also aim expanding the understanding of patient risk stratification and case selection, indicate using Logistic EuroScore and Society of Thoracic Surgeons Predicted Risk of Mortality score (STS-Score) to select suitable patients.

### 1.2 Objectives

The aim of this study is to provide an overview of TAVI registries and their reporting clinical outcomes based on the VARC-2 definitions. In addition, the comparability and adherence of VARC-2 reporting within the identified TAVI registries was reviewed.

## 2. Methods

### 2.1 Search methodology

The review was employed in line with the Preferred Reporting Items for Systematic Reviews and Meta-Analysis (PRISMA) guidelines [12]. The PubMed (Medline), the ScienceDirect, the Scopus database and the EMBASE were searched to identify all reports describing TAVI registries adapted to the VARC-2 definitions. The following search terms were used: “registry”, “Valve Academic Research Consortium” and “VARC”. Studies were also identified by scanning articles’ reference lists through citation snowballing, as well as gray literature searching.

### 2.2 Study selection

The articles describing TAVI registries adapting to the VARC-2 definitions were included in this review, additionally the TAVI registries were only for patients with aortic stenosis. Inclusion criteria and exclusion criteria for this review were listed in Table 1. No publication time restriction was used. The searching language was limited to English. The potential relevant title and abstract has been reviewed by two independent researchers after removing the duplicated studies. When studies presented duplicated patient cohorts, the most complete or updated reports will be selected.

**Table 1:** Inclusion criteria and exclusion criteria.

Inclusion criteria	Exclusion criteria
• TAVI registry studies for patients with aortic stenosis;	• Review, abstract, conference notice;
• Adapting to the VARC-2 definitions;	• Clinical studies;
• Peer-reviewed publications;	• Registry studies but not for TAVI;
• English language	• TAVI registry studies but not for patients with aortic stenosis;
	• Not under the VARC-2 definitions;
	• No complete description of clinical outcome following

### 2.3 Data extraction and quality of study assessment

Based on the VARC-2 clinical endpoint definitions, the following information was extracted from each registry’s publications [10,11]: patients’ characteristics and procedure characteristics including number of patients, type of device, access route, inclusion period, the Society of Thoracic Surgeons score (STS) (%), the Logistic EuroScore (%) and mean follow-up; 30-day clinical outcomes including: all-cause mortality, cardiovascular mortality (CV mortality), myocardial infarction (MI), stroke, bleeding, acute kidney injury (AKI), vascular complications and need for new permanent pacemaker (PPM); 1-year mortality; composited endpoints including: device success (72h), clinical efficacy (30 day), early safety (30 day) and time-related valve safety. The detail description of each major complication can be found in Kappetein et al. of guideline on using of VARC-2 definitions [11], which will be also briefly described in the result parts. Two researchers independently extracted data and rated the risk bias. The quality of observational studies included in our review was appraised by Newcastle-Ottawa Scale (selection, comparability, and outcome) criteria [13]. We assessed the possibility of publication bias both visually and formally to check if the publication contains description of each major complication based on VARC-2. For each subgroup of major complication, the authors list the major findings in the result part and analyze them in discussion part, respectively.

## 3. Results

## 3.1 Bibliographic research results

**Fig 1:**
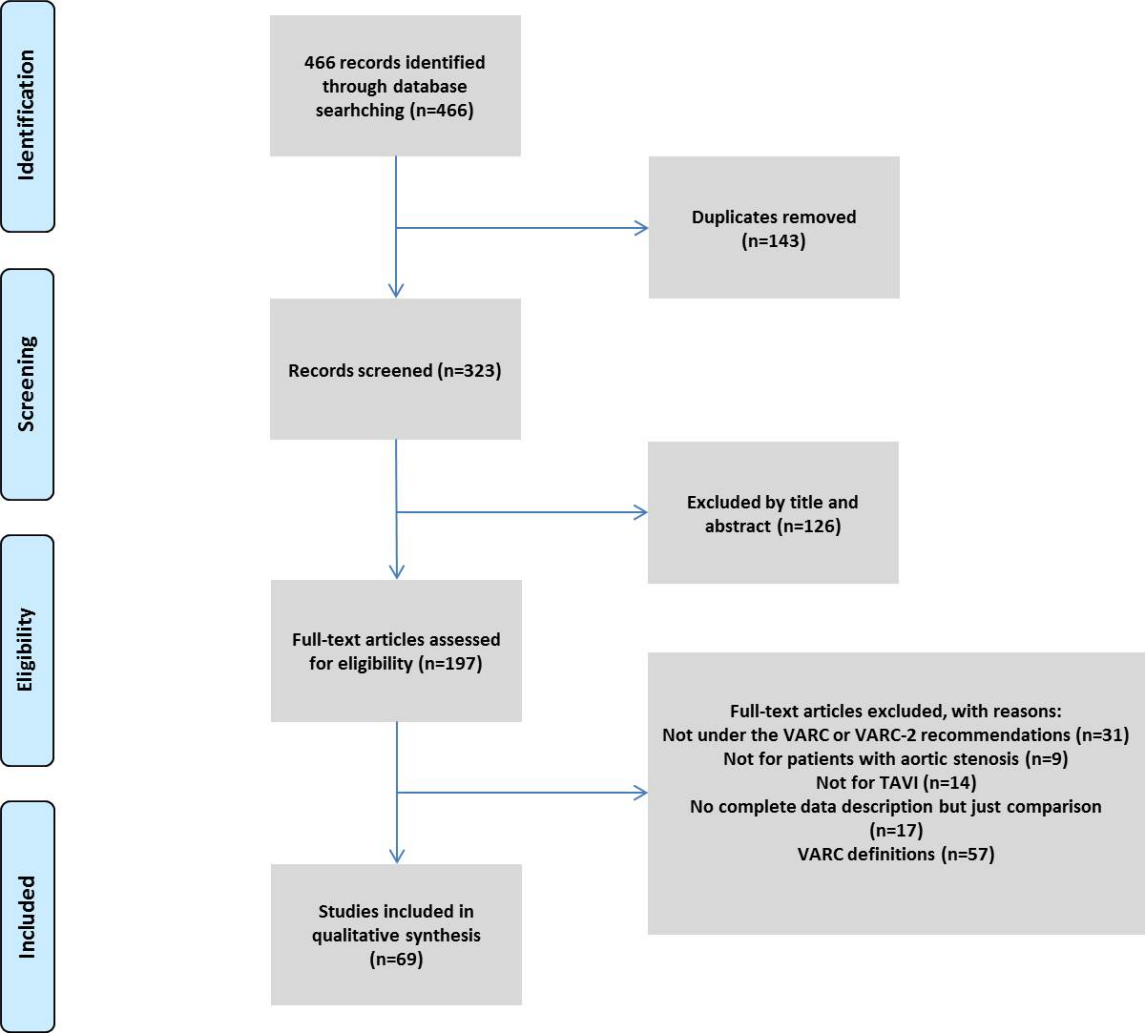
PRISMA Flow diagram of study selection.

This study identified 466 studies that were potentially relevant. Of all these studies, 37 originated from the PubMed (Medline) database, 274 from the Scopus database, 114 from the ScienceDirect and 41 were from the EMBASE. After removing duplicates, 323 abstracts have been reviewed independently by two researchers. According to the inclusion criteria and exclusion criteria, 197 studies from 74 registries were put into full text review. After full text reviewing, 69 studies from 20 registries were involved in the analysis in this study (Fig 1).

### 3.2 Study characteristics

20 TAVI registries reported VARC-2 definitions involved in our present review, the first publication of VARC-2 for TAVI registry study was published in 2013 [14]. The earliest inclusion time period was from 2005 to 2011 in the PRAGMATIC Multicenter Study [15]. Of all 20 registries, an overall sample size of 12,583 patients was involved in the present review. 6 registries with 4,607 patients (36.61%) used “Balloon-expandable SAPIEN Prosthesis” of Edwards Lifesciences (Irvine, California)[16–21], and one registry with 1,316 patients (10.46%) used “Self-expandable CoreValve Prosthesis” from Medtronic CoreValve (Minneapolis, Minnesota)[22], 10 registries with total 6,406 patients used both prostheses of SAPIEN Edwards and Medtronic CoreValve [23–31]. In addition, one registry with 154 patients used Lotus Valve System (Boston Scientific, MA) [32]; and one registry with 100 patients used a nonmetallic design TAVI system-Direct Flow Medical (DFM) TAVI system [33]. Transfemoral (TF) and transapical (TA) are two commonly used access routes in TAVI [34]. About 79.5% patients underwent TF access route, and the other 20.5% patients underwent non-TF route including TA, transaortic (TAo), subclavian (SC), transcarotid (TC), transaxillary (TAx) and direct aortic access route.

The results of our review are presented according to patients’ characteristics and procedure characteristics, mortality, each major complication category based on VARC-2 definitions in the following text. The pre-operative characteristics of patients from each registry are showed in Table 2, and the endpoints based on VARC-2 definitions for each registry are showed in Table 3.

**Table 2:** Study characteristics with patient characteristics and procedure characteristics.

Study	Type of device	Number of patients	Access Route	Inclusion period	STS (%)	Logistic EuroScore (%)	Mean Follow-up
Balloon-expandable Sapien Prosthesis Registry							
The ITER Registry **[20]**	SAPIEN XT 1303 (68.4), Edwards SAPIEN 601 (31.6)	1904	TF 1252 (65.8), TA 630 (33.1), TAo 18 (1), TAx 4 (0.2)	11.2007–12.2012	9.2±7.6	21.1±13.7	773 days*
The PARTNER II SAPIEN 3 Registry **[18]**	SAPIEN 3 THV	583 (HR/inoperable group)	TF 491 (84.2), TA 57 (9.8), TAo 35 (6.0)	10.2013–02.2014	8.7±3.7	-	-
	SAPIEN 3 THV	1078 (Intermediate risk group)	TF 952 (88.3), TA 81 (7.5), TAo 45 (4.2)	02.2014–12.2014	5.3±1.3	-	-
A Spanish single center TAVI Registry **[19]**	SAPIEN XT, SAPIEN	79	TF 64 (81), TA 15 (19)	06.2008–06.2012	5.9±2.9	16.9±9.1	28 m
The Swiss TAVI Registry **[17]**	Sapien 3	153	TF 133 (86.0)	02.-06.2014	7.1±6.5	23.7±15.9	30 days
	Sapien XT	445	TF 390 (87.6)	02.2011–01.2014	8.5±7.9	21.0±15.9	30 days
Rouen TAVI Registry **[16]**	SAPIEN XT 161 (68.2), Edwards SAPIEN	236	TF	05.2006–09.2012	-	20.6 ± 11.5	369 days
The SOURCE ANZ Registry **[21]**	SAPIEN	129	TF 67; TA 62	12.2008–12.2010	-	(26.8–28.8)*	1 year
Self-expandable CoreValve Prosthesis Registry							
The Italian CoreValve Registry **[22]**	CoreValve	1316	TF 1073 (81.6), SC 192 (14.6), TAo 50 (3.8)	06.2007–12.2012	-	20 (13–30)	13 m
Mixed Registry							
The WIN-TAVI Real-World Registry **[23]**	Mixed TAVI	1019	TF 923 (90.6), SC 26 (2.6), TA 26 (2.6), Tao 44 (4.3)	01.2013–12.2015	8.3±7.4	17.8±11.7	-
The Pooled-Rotterdam-Milano-Toulouse Registry **[30]**	Mixed TAVI	166	-	02.2014–05.2014	6.4 (4.0–11.5)	16.7 (11.2–26.6)	-
The Asian TAVR Registry **[31]**	Edwards SAPIEN; Medtronic CoreValve	848	TF 731, Non-TF 117	03.2010–09.2014	5.2±3.8	16.5±12.0	-
The Japan OCEAN TAVI Registry **[25]**	OCEAN-TAVI: Edwards SAPIEN XT	134	TF	10.2013–09.2014	-	18.8±13.3	1 year
Nassy database **[25]**	Nassy database: Edwards SAPIEN XT; Edwards SAPIEN 3; Medtronic CoreValve	178	TF	10.2013–09.2014	-	16.1±13.1	1 year
TAVI-Karlsruhe Registry **[28]**	Edwards SAPIEN/ SAPIEN XT THV n = 402; Symetis Acurate n = 11	1000	TA 413	05.2008–04.2012	-	24.3±16.2	1371 days
	Edwards SAPIEN /SAPIEN XT n = 399, Medtronic CoreValve n = 188		TF 587		-	22.2±16.2	
The Brazilian Registry **[24]**	CoreValve; Sapien XT	418	TF 402 (96.2), TAo 6 (1.2), TC 1 (0.2), SC 9 (2.2)	01.2008–01.2013	14±11	20.2±13.8	343.5 days
The PRAGMATIC Multicenter Study **[15]**	SAPIEN XT, SAPIEN, CoreValve	1062	-	11.2005–12.2011	8.7±6.5	22.5±13.3	2 years
Multicenter registry from North America, South America and Europe **[27]**	SAPIEN, SAPIEN XT, SAPIEN 3, CoreValve	1131	TF 73.1%, TA 20.3%, Tao 4.3%, SC 2.3%	03.2007–12.2014	8.2±6.8	-	21 m
The Royal Prince Alfred Hospital TAVI Program **[26]**	Edwards SAPIEN; Medtronic CoreValve	100	TF 68; TA 32	06.2009–07.2013	-	33.1±22.6	17 m
The University Hospital Zurich TAVI Registry **[29]**	Edwards SAPIEN 158 (45), Medtronic CoreValve 189 (54), Engager 3 (1)	350	TF 289 (83%)	05.2008–11.2012	-	22.1±13.8	389 days
Other TAVI Registry							
Nordic Lotus-TAVR registry **[32]**	Lotus Valve System	154	TF 151 (98.1), Direct aortic 3 (1.9)	-	5.0±2.8	-	-
DISCOVER Study **[33]**	Direct Flow Medical (DFM)	100	-	-	9.7±8.7	22.5±11.3	1 year

*medien follow-up

(26.8–28.8)*: the original data was taken from two groups

Tax = Transaxillary; Tao = Transaortic; SC = Subclavian; TC = Transcarotid

**Table 3:** Results of individual study based on VARC-2 Recommendations.

Study	All-cause mortality (30 days)	Cardiovascular mortality (30 days)	Mortality (1 year)	Myocardial infarction, n (%)	Stroke, n (%)	Bleeding, n (%)	Acute kidney injury, n (%)	Vascular access site and access-related complications, n (%)	Need for new PPM, n (%)
**Balloon-expandable Sapien Prosthesis Registry**									
The ITER Registry [20]	137 (7.2)	-	286 (15.0)	29 (1.5)*	54 (2.8)	499 (26.2)	155 (8.6)*	314 (16.5)	116 (6.1)
The PARTNER II SAPIEN 3 Registry **[18]**									
- HR/inoperable	13 (2.2)	8 (1.4)	-	3 (0.5)	12 (2.1)	117 (24.2)*	50 (7.6)	75 (12.9)	77 (13.3)
- IR	12 (1.1)	10 (0.9)	-	3 (0.3)	34 (3.2)	164 (15.2)*	56 (5.2)	131 (12.2)	109 (10.1)
A Spanish single center TAVI Registry **[19]**	(12.7)	(6.35)	(25.4)	2 (2.6)	2 (2.6)	9 (11.4)	14 (17.8)	12 (15.2)	3 (3.8)
The Swiss TAVI Registry **[17]**									
-Sapien 3	5 (3.3)	4 (2.6)	-	2 (1.3)	2 (1.3)	14 (9.2)	7 (4.6)	8 (5.2)	26 (17.0)
-Sapien XT	20 (4.5)	19 (4.3)	-	0 (0.0)	18 (4.0)	66 (14.8)	26 (5.8)	75 (16.9)	49 (11.0)
Rouen TAVI Registry **[16]**	11 (4.7)	-	(23.2)	-	-	18 (7.6)*	55 (23.3)	-33 (14.0)	-
The SOURCE ANZ Registry **[21]**	10 (7.8)	-	23 (17.8)	5 (3.9)	5 (3.9)	**-**	20 (15.6)	13 (10.1)	6 (4.7)
**Self-expandable CoreValve Prosthesis Registry**									
The Italian CoreValve Registry **[22]**	80 (6.1)	62 (4.7)	-	13 (0.9)	27 (2.0)	348 (26.4)	234 (17.8)*	93 (7.1)*	311 (23.6)
**Mixed Registry**									
WIN-TAVI Real-World Registry **[23]**	40 (3.4)	38 (3.3)	-	2 (0.2)	13 (1.3)	45 (4.4)*	13 (1.3)*	80 (7.7)*	118 (11.6)
The Pooled-Rotterdam-Milano-Toulouse Registry **[30]**	10 (6.0)	8 (4.8)	-	-	6 (3.6)	4 (2.4)*	12 (7.2)	8 (4.8)*	27 (16.2)
The Asian TAVR Registry **[31]**	21 (2.5)	14 (1.7)	81 (10.8)	-	32 (3.8)	92 (10.8)	28 (3.3)*	82 (9.7)	80 (9.5)
Inohara et al. 2016 (26)									
The Japan OCEAN TAVI Registry	0 (0)	0 (0)	-	2 (1.5)*	2 (1.5)	23(17.2)	2 (1.5)*	17(12.7)	8 (6.0)
Nassy database	1 (0.6)	1 (0.6)	-	0 (0)*	1 (0.6)	30(16.8)	2 (1.1)*	27(15.2)	39 (21.9)
TAVI-Karlsruhe Registry **[28]**									
-TA	(6.1)	(4.1)	-	(2.7*)	(1.7)	(28.8)	(35.1)	(2.9)	(10.7)
-TF	(6.5)	(5.1)	-	(1.7*)	(2.3)	(28.6)	(19.9)	(19)	(15.7)
The Brazilian Registry **[24]**	(9.1)	(7.9)	(21.5)	(0.7)	(3.5)	(18.5)	(20.0)	(13.8)	(24.4)
PRAGMATIC Multicenter Study **[15]**	63 (5.9)	56 (5.3)	187 (18.5)	12 (1.1)*	42 (4)	460 (43.3)	257 (24.2)	227 (21.4)	165 (15.6)
Multicenter registry from America and Europe **[27]**	65 (5.7)	-	-	-	40 (3.5)	57 (5.0)*	-	136 (12.0)*	173 (15.3)
The Royal Prince Alfred Hospital TAVI Program **[26]**	3 (3.0)	2 (2.0)	7 (7.0)	2 (2.0)*	4 (4.0)	30 (30.0)	16 (16.0)	17 (17.0)	13 (13.0)
The University Hospital Zurich TAVI Registry **[29]**	32 (9.1)	31 (8.7)	(21.0)	(2.0)	(2.9)	(4.6)	(5.7)	(7.4)	(18.9)
**Other TAVI System**									
Nordic Lotus-TAVR registry **[32]**	3 (1.9)	-	-	-	5 (3.2)	3 (1.9)*	2 (1.3)*	4 (2.6)*	43 (27.9%)
DISCOVER Study **[33]**	1 (1.0)	1 (1.0)	10 (10.0)	1 (1.0)	7 (7.0)	9 (9.0)*	1 (1.0)*	13 (13.0)	17 (17.0)

29 (1.5)*, 2 (1.5)*, 0 (0)*, (2.7*), (1.7*), 12 (1.1)* and 2 (2.0)* in Myocardial infarction list are for Periprocedural MI (<72h)

In the bleeding list, 117 (24.2)*, 164 (15.2)* and 57 (5.0)* are for Life-threatening/disabling and Major bleeding; 18 (7.6)*, 45 (4.4)*, 4 (2.4)* and 3 (1.9)* are for Life-threatening bleeding; 9 (9.0)* is for Major bleeding

In the AKI list, 155 (8.6)*, 13 (1.3)*, 28 (3.3)* and 2 (1.3)* are for Acute kidney injury, stage 2 or 3; 234 (17.8)*, 2 (1.5)*, 2 (1.1)* and 1 (1.0)* are for Acute kidney injury Stage 3

In vascular complications list, 93 (7.1)*, 80 (7.7)*, 8 (4.8)*, 136 (12.0)* and 4 (2.6)* are for Major vascular complications

#### 3.2.1 Pre-operative characteristics

Logistic EuroSCORE (LES) and Society of Thoracic Surgeons Predicted Risk of Mortality (STS-PROM) score are commonly used in clinical trials to identify high-risk surgical or “inoperable” patients for TAVI [35]. The logistic Euroscore calculated by means of a logistic regression equation, ranges from 0 to 100%, with high scores indicating greater risks and a score of more than 20% indicating high surgical risks [36]. In addition, the STS score >8 is defined as “high risk”, 4–8 is defined as “Moderate risk” [11]. 12 registries in our review presented the STS score, the mean STS score ranged from 5.2% in the Asian TAVR Registry to 14% in the Brazilian Registry [24,31]. 18 out of 20 registries in our review reported the logistic EuroScore, the mean Logistic EuroScore ranged from 16.1% in Nassy Database to 33.1% in the Royal Prince Alfred Hospital TAVI Program [25,26]. The shortest mean follow-up period was in the Swiss TAVI Registry, which was 30 days [17]. The longest mean follow-up period was in the TAVI-Karlsruhe Registry, which was 1,371 days [28]. All other registries have a mean follow-up over 1 year.

#### 3.2.2 Mortality

According to the VARC-2 definitions, “all-cause, cardiovascular, and non-cardiovascular mortality should be reported after 30 days during the follow-up [11].” The 30-day all-cause mortality ranged from 0 in OCEAN-TAVI Registry to 12.7% in a Spanish single center TAVI Registry [19,25]. In the present review, from 20 registries, 14 registries reported the CV mortality at 30 days. The ratio of CV mortality in all-cause mortality at 30 days ranged from 50% in a Spanish single center TAVI Registry to 96.8% in the University Hospital Zurich TAVI Registry [19,29]. In addition, 10 registries reported 1-year all-cause mortality. From most of the registries, the all-cause mortality at 1 year was 2 to 3-fold higher than that at 30 days. However, the 1 year all-cause mortality in the Rouen TAVI Registry was 5-fold higher than their 30 days all-cause mortality [16]; and in the DISCOVER Study, it was 10-fold higher [33].

#### 3.2.3 Myocardial infarction

VARC-2 recommends the systematic collection of biomarkers of myocardial injury prior to the procedure, within 12–24 hours after the procedure, at 24 hours thereafter, at 72 hours or at discharge [11]. The definition of periprocedural MI will be based on a combination of clinical criteria and cardiac biomarkers within 72 hours following TAVI [11]. 9 registries reported MI rate based on VARC-2 definitions, and 7 registries reported peri-procedural MI rate (<72h). In our review, most of registries presented MI rates ranging from 0.5% to 2%. The SOURCE ANZ Registry reported a higher MI rate of 3.9%. The TA-TAVI group with 62 patients in this registry achieved an even higher MI rate, was 6.45% [21]. The similar results could be found in the TAVI-Karlsruhe Registry, where peri-procedural MI occurred in 2.7% of 413 patients underwent TA route [28].

#### 3.2.4 Stroke

VARC-2 recognizes that an assessment of stroke is incomplete without an appropriate measurement of the disability resulting from the stroke. VARC-2 now recommends the use of terms “disabling” and “nondisabling” [11]. In the present review, 19 registries report all including disabling stroke, non-disabling stroke and TIA based on VARC-2 definitions. It ranged from 0.6% in Nassy Database to 7% in a single registry [25,33].

#### 3.2.5 Bleeding

VARC definition of bleeding complications is divided into life-threatening or disabling bleeding, major bleeding, and minor bleeding [10,11]. For the present review, 14 registries reported all bleeding complications, 18 registries reported life-threatening bleeding at 30 days after TAVI, and 17 registries reported major bleeding. The overall bleeding rate ranged from 4.6% in the University Hospital Zurich TAVI Registry [29] to 43.3% in the PRAGMATIC Multicenter Study [15].

#### 3.2.6 Acute kidney injury (AKI)

VARC-2 recommends diagnosing AKI at 30 days based on increasing in serum creatinine and urine output, which can be categorized into stages 1 to 3. Stage 3 indicates the most serious level. 19 registries reported AKI complications, most of them having rates of AKI with 2.0% to 3.0%. However, in the ITER Registry and the PRAGMATIC Multicenter Study, *AKI stage 2 to stage 3 at 30 days* occurred in 8.14% and 9.23% patients, respectively [15,22].

#### 3.2.7 Vascular complications

VARC-2 lists major and minor vascular complications. 19 registries reported the major vascular complications, ranged from 2.42% in the TA group of the TAVI-Karlsruhe Registry [28], to 17.54% of patients undergoing TF route in the TAVI-Karlsruhe Registry [28].

#### 3.2.8 New pacemaker implantation

VARC-2 proposes the systematic collection of data on the frequency of implant-related new permanent pacemaker implantation [11]. 18 registries reported the patient number in which implanted a new pacemaker. The rate from pure Edwards registries ranged from 3.8% to 17.0% [17,19]. In the pure CoreValve registry, 23.63% of patients needed a new pacemaker after TAVI [22]. In addition, both of the new Lotus Valve System and Direct Flow Medical (DFM) system had a higher requirement of pacemaker, with a rate of 27.9% and 17.0%, respectively.

#### 3.2.9 Composite endpoints

The composite endpoints according to VARC-2 definition included: (1) device success, (2) early safety at 30 days, (3) clinical efficacy after 30 days and (4) time-related valve safety. *Device success* indicates the absence of procedural mortality, correct positioning of a single prosthetic heart valve as well as intended performance of the prosthetic heart valve. *Early safety* is a combined endpoint at 30 days including all-cause mortality, all stroke, life-threatening bleeding (LT bleeding), AKI-stage 2 or 3, coronary artery obstruction requiring intervention, major vascular complication and valve-related dysfunction requiring repeat procedure. *Clinical efficacy* is a combined endpoint after 30 days including all-cause mortality, all-stroke, requiring hospitalizations for valve-related symptoms or worsening congestive heart failure, NYHA class III or IV as well as valve-related dysfunction. *Time-related valve safety* combines valve dysfunction, endocarditis, and thrombotic complications of the prosthesis. In our review, 13 studies reported device success rate, which ranged from 76.3% to 97.8% [15,16,19,20,22,25–27,29,31,33,37]. 11 studies reported early safety rate, but it ranged from 10% to 92.2% [15,19,20,23–26,29,31–33]. Only three registries reported clinical efficacy [19,20,32]. There is no registry reported time-related valve safety. Detail information for each registry was described in Table 4.

**Table 4:** Composite endpoints from individual studies based on the VARC-2.

Study	Device success	Clinical efficacy (30 day)	Early safety (30 day)
**Balloon-expandable Sapien Prosthesis Registry**			
The ITER Registry **[20]**	88.1	1030 (54.1)	1418 (74.5)
The PARTNER II SAPIEN 3 Registry **[18]**	-	-	-
A Spanish single center TAVI Registry **[19]**	69 (87.3)	52 (65.8)	55 (69.6)
The Swiss TAVI Registry **[17]**	-	-	-
-Sapien 3	-	-	-
-Sapien XT	-	-	-
Rouen TAVI Registry **[16]**	219 (92.8)*	-	-
The SOURCE ANZ Registry **[21]**	**-**	-	-
**Self-expandable CoreValve Prosthesis Registry**			
The Italian CoreValve Registry (22)	1,241 (94.7%)	-	-
**Mixed Registry**			
WIN-TAVI Real-World Registry **[23]**	-	-	147 (14.0)
The Pooled-Rotterdam-Milano-Toulouse Registry **[30]**	-	-	-
The Asian TAVR Registry **[31]**	725 (85.5)	-	124 (14.6)
Inohara et al. 2016 (26)			
The Japan OCEAN TAVI Registry	131 (97.8)	-	13 (9.7)
Nassy database	174 (97.8)	-	20 (11.2)
TAVI-Karlsruhe Registry **[28]**	-	-	-
The Brazilian Registry **[24]**	319 (76.3)	-	22.7
PRAGMATIC Multicenter Study **[15]**	974 (91.7)	-	308 (29)
Multicenter registry from America and Europe **[27]**	879 (78.8)	-	-
The Royal Prince Alfred Hospital TAVI Program **[26]**	94.0	-	86 (86)
The University Hospital Zurich TAVI Registry **[29]**	88.0	-	67 (19.1)
**Other TAVI Registry**			
Nordic Lotus-TAVR registry **[32]**	-	141 (91.6)	142 (92.2)
DISCOVER Study **[33]**	91.0	-	10.0

*device failure rate in this study is 7.2%.

## 4. Discussion

To our knowledge, this is the first systematic review analyzing clinical outcome reporting based on VARC-2 definitions. TAVI has become the fastest growing cardiac procedure since the introduction of coronary stents, with penetration rates of over 35% in countries such as Switzerland and Germany [38], where this rate has achieved to 52% In 2015 [39]. Despite this growth, there was a lack of standardized reporting on clinical outcomes for patients undergoing TAVI before the introduction of VARC and VARC-2 definitions. As demonstrated and confirmed by Genereux et al. in a pooled analysis of 3,519 patients, VARC definitions already represent a new standard for consistency in reporting clinical outcomes for patients undergoing TAVI [40]. The VARC-2 is an updated version of the original VARC. In the results section above, the authors summarized the registries reports using VARC-2 definitions based upon mortality as well as major complication categories. To address and support patients’ safety and procedural quality as demanded by regulatory bodies and HTA agencies, three main parts are needed to be taken into discussion: first of all, the authors provide the summary of the proportion use VARC-2 overall all TAVI registries; and investigate the trends of research in the field of TAVI since the introduction of VARC definitions; Secondly, the authors highlight important notices of reporting status for each complication, meanwhile, the authors compare the registry outcomes with meta-analysis of RCTs which use VARC and VARC-2 definitions [41], as well as the meta-analysis of studies based on VARC definitions [40]. The authors also provide the influence factors description through comparison of single registry for each complication. In the last part, the author will summarize some aspects needed to be updated from VARC-2 definitions.

### 4.1 Summary of proportion use VARC-2 overall TAVI registries

During the search period, there are 44 registries in total potentially identified. 50% of them followed the VARC-2 definitions, and 13 used VARC definitions, additionally 10 registries did not follow both VARC definitions. Comparing the clinical outcome reporting system between the registries who followed the VARC definitions and who did not, the author clearly found that the one followed VARC definition have more structured reporting system. To be noticed, the reporting system is not the factor to influence clinical outcome. The biggest advantage of uniform and structured reporting system is to help comparing clinical outcome among different registries or study groups. The registries that did not follow VARC definition report clinical outcome mostly based on patients’ medical records; as a result, the clinical outcome in these registries can be only compared with their own patients’ baseline characteristics. It is impossible to compare clinical outcome with other registries. Another notice from the registries not following VARC definitions is that these registries do not separate cardiovascular mortality from all-cause mortality, which may be misleading, resulting in disproportionate reporting of mortal events unrelated to either the treatment device or the procedure [10,11].

Complications are defined uniformly in all studies using VARC definitions, which make clinical outcome comparison available. Available comparison gives the researchers a chance to do systematic review as well as meta-analysis. This point can be proved by the number of systematic review and meta-analysis in the field of TAVI. The authors make a brief search in PubMed (Medline) database for the number of systematic review (SR) and meta-analysis (MA) in the field of TAVI. The result proved our hypothesis. We found one SR in 2010 and 2011, respectively; one SR and two MA in 2012; four MA were conducted in 2013, which started to mention VARC definitions in the result part; there were eight SR and MA published in 2014; the number of published SR and MA in 2015 and 2016 is 16, 10, respectively. The trend of formulating a SR or MA is from focusing one complication to all complications after the introduction of VARC definitions; in addition, the study design is changing from based on medical records, to analyze the result according to VARC definitions, to just reviewing the studies reporting clinical outcome based on VARC definitions.

### 4.2 Single complication reporting needs to be noticed

The result part illustrates how TAVI registries report clinical outcome according to VARC-2 definition for mortality and each complication. In the following text the author will discuss what should be notified when report mortality and major complications according to VARC-2 definition. In addition, although the result summarizing from each single studies is not the priority focus of our review, the results comparison can show if the reporting system influences the clinical outcome, and which aspects can influence single complication. In this part, the author will compare the result gotten from our review to the meta-analysis based on VARC definitions and based on RCTs, then to highlight the general accepted influence factors for each complication.

#### 4.2.1 Mortality

The reporting of *CV mortality at 30 days* and *immediate procedural mortality* is vital to the cumulative clinical experience. Therefore, VARC-2 recommends the collection of *immediate procedural mortality* data to capture intra-procedural events that result in immediate or consequent death within 72 hours post-procedure [11]. However, there is no registry to report *immediate procedural mortality* data of all included studies, and only 14 registries reported *CV mortality at 30 days*. It is already stated in the VARC definitions that it is essential to report CV mortality to reduce disproportionate reporting of mortal events unrelated to either the treatment or to the procedure [10].

Because there may be either under-reporting of early device failure or unknown status at early report times, VARC definitions are suggesting *all-cause mortality at 1 year* as a primary endpoint for TAVI clinical trials [10]. In our review, only 10 registries reported *1-year all-cause mortality*. To increase patients’ safety and avoid under-reporting of early device failures, registries should focus on long-term follow up, especially 1-year clinical outcomes. Outcome reporting should be established for both at 30-day as well as at 1-year. If the mortality rates are significantly different when comparing 30-day and 1-year data, the registry should list and clarify the cause of death.

In our review the *30-day all-cause mortality* ranged from 0 to 12.7% [19,25]. Eight studies from our review reported a rate of *30-day all-cause mortality* about 6.0% [41]. The 30-day all-cause mortality from a pooled analysis of studies based on VARC is 7.8% [40], which is comparable with the result from our study; the result from 5 RCTs is 3.7% [41], which is better than the result from our study, main reason is that the patients selection from for RCTs is lower risk and have a better prognosis. The patient selection is one of the influence factors of mortality, especially cardiovascular mortality. In addition, De Brito et al. demonstrated that the training program and supervision provided to centers can reduce the mortality rate [24]. It could be confirmed that the rate of mortality was impacted by the program volume, with the risk of death being 2.5 times greater in low volume centers [27]. Low hospital volume status was associated with significantly greater odds of death, bleeding, and MI, which was demonstrated by a study from the US national representative database [42].

#### 4.2.2 Myocardial infarction (MI)

It is recommended that MI reporting differentiate between periprocedural MI (<72h) and spontaneous MI (>72h) [11]. VARC-2 recommends the systematic collection of biomarkers of myocardial injury prior to the procedure, within 12–24 hours after the procedure, at 24 hours thereafter, at 72 hours or at discharge [11]. However, in our review, we identified only 11 registries who reported periprocedural MI. Most of registries presented MI rates ranging from 0.5% to 2% in present review. A similar result can be also found in Genereux et al. based on VARC definitions, of 1.1% [40]. The result from 5 RCTs has the same MI rate, which is 1.1% [41]. The SOURCE ANZ Registry reported a higher MI rate of 3.9%, the TA-TAVI group with 62 patients in this registry achieved an even higher MI rate with 6.45% [21]. Similar results could be found in the TAVI-Karlsruhe Registry, where periprocedural MI occurred in 2.7% of the 413 patients who underwent TA route [43]. It might be argued that this high number of MI is due to TA routes. However, this is not reflected by a meta-analysis presented by Panchal et al., that the incidence of MI was not statistically significant between the TF and TA TAVI groups [44]. Besides the access route, sex difference might be an influence factor of having post-MI, the WIN-TAVI Real-World Registry [23] with a group of female patients had very low MI rate (0.2). In addition, the development of device and TAVI procedure also help to reduce the rate of MI, which showed in the Sapien XT group in the Swiss TAVI Registry [17], which had a rate of post-MI of 0.

#### 4.2.3 Stroke

According to VARC-2 definitions, the *all-stroke rate* includes TIA, disabling stroke and non-disabling stroke [11]. 19 registries reported *all-stroke rate*, which ranged from 0.6% to 7% in the present review [25,33]. The *all-stroke rate* reported by Muralidharan et al. was 3.07% in 29,043 patients [45]. The rate of all neurological events estimated by Genereux et al. was 5.7% [40]. The result from 5 RCTs is 5.3%, which has no big difference with the result from registries [41]. Siemieniuk et al. demonstrated that TA-group patients had a higher incidence of stroke occurrence, with 6.5% higher than that of TF-group patients [46]. However, a single center study from our review displayed different results, which showed a stroke incidence within the TF group is higher than that within the TA group (2.3% vs. 1.7%) [43]. In addition, older patients may have a higher risk of stroke than younger patients. If patients have better baseline characteristics, they will have a better outcome. For example, the logistic EuroScore for Nassy database is 16.1±13.1, just one person has stroke afterwards [25]. *Perioperative stroke* is closely related to mortality rate. Muralidharan et al. reported *30-day stroke-related mortality rate* of 12.27%, which is 4 times higher for patients with *perioperative stroke* in comparison to patients without *perioperative stroke* (28.22% vs. 6.40%).

#### 4.2.4 Bleeding

Bleeding complications should be reported in three categories: life-threatening or disabling bleeding, major bleeding and minor bleeding [11]. The reporting situation of bleeding rate is satisfactory in our review. The authors can identified both *life-threatening bleeding* and *major bleeding* from 18 registries. The overall bleeding rate ranged from 4.6% in the University Hospital Zurich TAVI Registry [29] to 43.3% in the PRAGMATIC Multicenter Study [15]. In the PRAGMATIC Study, 19.1% of patients experienced *major bleeding* [15]. Genereux et al. reported *major bleeding rate* of 22.3%, and *life-threatening bleeding rate* of 15.6% [40]. In addition, 5 RCTs reported a major bleeding rate of 16.4% [41]. Siemieniuk et al. reported that bleeding was reduced with use of both TF-TAVI (25.2%) and TA-TAVI (19.4%) as compared with SAVR [46]. This result is in line with the result of a single study from our review, which showed a similar result from both TF and TA groups (28.6% vs. 28.8%) [43].

#### 4.2.5 Acute kidney injury (AKI)

There are 3 stages of AKI based on VARC-2 definitions: stages 1, 2, and 3. In our review, the rate of *AKI stage 2* and *AKI stage 3* can be only identified from 8 registries. Most registries have a rate of *AKI stage 2 to 3* with 2.0% to 3.0% in our review. The ITER Registry had a higher rate of 8.14% of *AKI stage 2 to 3*, also with a higher rate of *life-threatening* and *major bleeding* of 20.53% [20]. A similar result can be found in the PRAGMATIC Multicenter Study, which reports a rate of *life-threatening combined with major bleeding*, *AKI stages 2 to 3*, 32.20% and 9.22% respectively [15]. The interaction between *bleeding* and *AKI* in TAVI patients remains unclear, and should be investigated in future studies. The pooled estimate rate of *AKI stages 2 to 3* by Genereux et al. from 1,275 patients was 7.5% [40]. The rate of AKI from the meta-analysis of 5 RCTs is 2.5% [41]. Siemieniuk et al. showed a higher risk of TA-TAVI than TF-TAVI when receiving AKI post procedure [46]. This is also demonstrated by Schymik et al., who report that the TF group had a lower rate of AKI than TA group (19.9% vs. 35.1%) [43].

#### 4.2.6 Vascular complications

Vascular complications can be categorized into two types: major vascular complication and minor vascular complication. All of the identified registries have reported *vascular complication* rates in our review. Most studies in our review reported *vascular complications* ranging from 3.7% to 9.8%. *Major vascular complications* occurred in 11.9% of patients as reported by Genereux et al. [40]. The vascular complications rate from RCTs is 7.9%, which is better than the result from registries [41]. Patients were more likely to experience *vascular complications* following TAVI compared to SAVR [41,46]. The TF-TAVI group of 587 patients in the TAVI-Karlsruhe Registry had a higher *vascular complication* rate with 17.55% [28], the result from their TA group was 2.42% [28]. It is generally accepted, that the TA approach has been associated with a lower rate of *vascular complications* than the TF route [47–49]. The author assumed that the influence factor of patients having vascular complications after TAVI have highly relevant to the access route, but not the baseline characteristics, like the logistic EuroScore for Nassy database is 16.1±13.1, there are still 15.2% patients in this registry have vascular complications afterwards [25].

#### 4.2.7 New pacemaker implantation

VARC-2 proposes the systematic collection of data on the frequency of implant-related new permanent pacemaker implantation [11]. The reporting outcome can be identified from all the registries in our review. The overall *new pacemaker implantation rate* in our review is similar to the previous meta-analysis, where the rate of new pacemaker implantation was 13.9% [40]. The result is also similar to the result taken from RCTs, which is 14.4% [41]. New permanent pacemaker implantation was more common with TAVI than SAVR [41,46]. It is generally accepted that the self-expandable CoreValve has a higher rate of new pacemaker requirement than Edwards valve [40]. This can be reflected from our review, the rate from pure Edwards registries ranged from 3.8% to 17.0% [17,19], and 23.63% of patients needed a new pacemaker after TAVI in the pure CoreValve registry [22]. And it is showed in Nordic Lotus-TAVR registry [32] and DISCOVER Study [33], the new device has also a higher rate of new pacemaker requirement than Edwards valve.

#### 4.2.8 Composite endpoint

VARC-2 definitions recommended composite endpoints for TAVI safety and effectiveness [10,11]. However, in our study, the reporting status from identified registries was disappointing. For example, 11 studies reported an *early safety* rate, but it ranged from 10% to 92.2%, only three registries reported a rate of *clinical efficacy*, and no registry report a rate for *time-related valve safety*. This lack of data could be because the registry groups misunderstand the calculation of composite endpoint, or that they do not have any data to report.

### 4.3 Some aspects needed to be updated from VARC-2 definitions

The reason why TAVI registries not following VARC definitions are mainly because that the time they collected data was before the introduction of the VARC definition. The other reason is because the registry aimed to investigate some special complications after undergoing TAVI, one study performed by the GAMES database aimed to describe the characteristics of infective endocarditis after undergoing TAVI[50]; the multicenter German TAVI registry aimed to predict the relations between mortality and aortic regurgitation after TAVI [51]. This can be also reflected from the current systematic review and meta-analysis, which aimed to investigate special complications like endocarditis, cognitive functions, leak post-transcatheter as well as quality of life, did not require included studies designing based on VARC definitions. The registries not following VARC definition also indicate that medical procedure and experience are developing; a standard recommendation needs to be updated to fulfill the development of medical technology and clinical experience.

### 4.4 Study limitations

There are several limitations in this review. First of all, the authors were only able to review articles in English. Therefore, some important article could be missing. In addition, this review focused on major complications based on VARC-2 definitions. The authors did not analyze other complications, which could also influence the result.

## 5. Conclusion

VARC and VARC-2 definitions are more and more widely used in clinical studies as well as in registry studies. However, since their introduction in 2011, VARC definitions are still not systematically reported in TAVI studies. These endpoint definitions warrant a concise and systemic analysis of outcome measures in high-risk patient populations. These standardized endpoint definitions make study result comparisons feasible, providing better insights by differentiating products and approaches, and thus increasing transparency for patients.

For regulatory bodies, standardized reporting based on VARC-2 can support post-market surveillance of already approved TAVI products to monitor safety and to react to adverse events timely. For HTA agencies VARC-2 definitions allow pooled analyses of various studies, and thus provide sufficient evidence on cost and effectiveness of different types of products and different access routes. Taking VARC-2 definitions into account, payers can choose the most appropriate procedure for different patient groups, making TAVI more effective and less costly.

## Supporting information

S1 Table PRISMA checklist. This manuscript is a systematic review in line with PRISMA checklist.
(DOC)
